# Analysis of mutations of defensin protein using accelerated molecular dynamics simulations

**DOI:** 10.1371/journal.pone.0241679

**Published:** 2020-11-30

**Authors:** Bharati Pandey, Chetna Tyagi, Gopal Kumar Prajapati, Awdhesh Kumar Mishra, Abeer Hashem, Abdulaziz A. Alqarawi, Elsayed Fathi Abd_Allah, Tapan Kumar Mohanta

**Affiliations:** 1 School of Biotechnology, Jawaharlal Nehru University, New Delhi, India; 2 Department of Microbiology, Faculty of Science and Informatics, University of Szeged, Szeged, Hungary; 3 Department of Bio-Engineering, Birla Institute of Technology, Mesra, Ranchi, Jharkhand, India; 4 Department of Biotechnology, Yeungnam University, Gyeongsan, Gyeongbuk, Republic of Korea; 5 Botany and Microbiology Department, College of Science, King Saud University, Riyadh, Saudi Arabia; 6 Mycology and Plant Disease Survey Department, Plant Pathology Research Institute, ARC, Giza, Egypt; 7 Plant Production Department, College of Food and Agricultural Sciences, King Saud University, Riyadh, Saudi Arabia; 8 Natural and Medical Sciences Research Center, University of Nizwa, Nizwa, Oman; University of Michigan, UNITED STATES

## Abstract

Plant defensins possess diverse biological functions that include antifungal and antibacterial activities and α-amylase and trypsin inhibitory properties. Two mutations, G9R and V39R, were confirmed to increase the antifungal activity of *Raphanus sativus* antifungal protein 2 (RsAFP2). Accelerated Molecular Dynamics (aMD) were carried out to examine the conformational changes present in these RsAFP2 mutants, and its two closest homologs compared to the wild-type protein. Specifically, the root mean square fluctuation values for the eight cysteine amino acids involved in the four disulfide bonds were low in the V39R mutant compared to the wild-type. Additionally, analysis of the free energy change revealed that G9R and V39R mutations exert a neutral and stabilizing effect on RsAFP2 conformation, and this is supported by the observed lower total energy of mutants compared to the wild-type, suggesting that enhanced stability of the mutants. However, MD simulations to a longer time scale would aid in capturing more conformational state of the wild-type and mutants defensin protein. Furthermore, the aMD simulations on fungal mimic membranes with RsAFP2 and its mutants and homologs showed that the mutant proteins caused higher deformation and water diffusion than the native RsAFP2, especially the V39R mutant. The mutant variants seem to interact by specifically targeting the POPC and POPI lipids amongst others. This work highlights the stabilizing effect of mutations at the 9^th^ and 39^th^ positions of RsAFP2 and their increased membrane deformation activity.

## 1. Introduction

Plants have evolved natural resistance to abiotic and biotic stresses, such as adverse environmental conditions and infection by microbial and insect pathogens. The cell wall of plants functions as a physical barrier and provides the first line of defense [1]. Additionally, plants produce several specialized metabolites to protect against infections or physical damage. The most prominent of these are the antimicrobial peptides (AMPs) that function as a component of the innate immune system and are essential for protecting plants from microbial invaders [2, 3]. Defensins form a family of AMPs that are broadly dispersed among eukaryotes. Plant defensins (formerly known as γ-thionins) possesses different structural configuration (β1-α-β2-β3) from mammalian defensins (α-β1-β2-β3). They are small cationic peptides consisting of 45-54 amino acids that comprise four to five intramolecular disulfide bonds [4–6]. These defensins exhibit diverse biological functions that include antifungal [7–12] and antibacterial [13, 14] activities, and α-amylase and trypsin inhibitory properties [15, 16]. In addition to their antimicrobial roles, plant defensins are also involved in the biotic stress response and in plant growth and development [17]. There is currently a great deal of interest in defensins due to their broad-spectrum antibacterial and antifungal activities [18]. These cationic proteins need to disrupt the outer membrane of the host to gain access intracellular targets. However, AMP show diverse mechanism of actions including anionic lipid charge clustering [19], toroidal pore formation [20], adopt specific structure conformation corresponding to the outer membrane (like MSI-594 AMP) [21]. It is reported that palmitoylated bovine-β-defensin-2 (BNBD-2) interact more efficiently with membrane in presence of NaCl, thus improving the antibacterial activity [22]. In a recent study [23], demonstrated that peptide chain length and conformation locking are very critical parameters while designing potent novel antimicrobial peptide.

Plant defensins have high diversity in amino acids sequences composition, only few residues (eight cysteine (C), two glycine (G) and one aromatic amino acid were conserved) [24]. However, they share a common 3-dimensional structure comprised of three antiparallel β-sheets and one α-helix, stabilized by disulfide bridges of conserved cysteine residues [4]. More than 300 plant defensins from different plant species have been reported and their annotation can be found in the NCBI. These proteins specifically interact with fungal membrane lipid to exert their effect. Several three-dimensional structures of these defensin proteins have been submitted to the PDB, such as RsAFP2 from *R*. *sativus* (PDB ID, 2N2R); [25], OsAFP1 from *Oryza sativa* japonica (PDB ID, 6LCQ); [26] and OsAFP1 from *Heuchera sanguinea* (PDB ID, 2N2Q); [27].

Two new classes of antifungal proteins, *R*. *sativus* antifungal protein 1 (RsAFP1) and *R*. *sativus* antifungal protein 2 (RsAFP2), have previously been isolated from radish seed. Both proteins have only two amino acid differences in their sequences (Q5E and R27N) [25]. The residues, Thr-10, Ser-12, Leu-28, Tyr-38, Phe-40, Ala-42, Lys-44, Ile-46 and Phe-49 are important for the antifungal activity of RsAFP2 [25]. They exhibit highly potent antifungal activity against diverse pathogenic fungi, including those that affect plants and humans (*Candida albicans*). A substantial decrease in the anti-fungal activity was observed for two AF2 variants, RsAFP2 (L28R) and RsAFP2 (I46R), and a two-fold increase in the uptake of Ca^2+^ in response to *Fusarium culmorum* infection was observed for the RsAFP2 (G9R) and RsAFP2 (V39R) mutants [25]. The IC50 value of RsAFP2 against *Fusarium culmorum* in synthetic low ionic strength medium (SMF2) and the same medium supplemented with 1 mM CaCl2 and 50 mM KCl was found to be 3.3±0.6 and 3.2±0.3 μg/ml for G9R and V39R, respectively [25].

Molecular dynamics (MD) technique has emerged as an increasingly prevalent tool that can be used to analyze the atomic movement and flexibility in proteins, peptide protein-ligands, and protein-DNA complexes within a box filled with water and ions [28–30]. Recently, the effect of a disulfide bond present in an antifungal peptide derived from *Petunia Hybrida* was analyzed using 5 ns MD simulations [31]. To investigate the role of mutations in the RsAFP2 in increasing peptide stability, we performed accelerated Molecular Dynamics (aMD) simulations in water and lipid bilayer. aMD simulations is an enhanced sampling technique that works by decreasing the energy barriers between different states of a biological system [32]. This fastens the process of observing all possible states of the target system. In other words, a 500 ns long aMD simulation covers the same energy landscape equivalent to 1 ms of classical MD simulation. Therefore, in this study we used aMD for better sampling of the phase space of RsAFP2 protein variants and their interaction with the fungal membrane mimics.

## 2. Material and methods

### 2.1. Multiple sequence alignment

The three-dimensional structure (3D) of the antifungal peptide isolated from *R*. *sativus* L. (radish) was downloaded from PDB (PDB ID, 2N2R) [33]. The structural information of the antifungal protein was solved using Nuclear Magnetic Resonance [34]. The length of the anti-fungal peptide was 51 amino acid residues, and these were used as a query to search against the non-redundant protein database using protein BLAST [35]. The hits following the selection criteria of E-value ≤ 10^-20^, sequence identity ≥ 30%, query coverage ≥ 70%, and the complete sequence were considered for further analysis. The selected sequence was aligned using Clustal omega based on the HMM profile-profile method [36]. Two closet homologs of RsAFP2 protein from *Brassica oleracea* (named as homolog-1 or HM1) and *Arabidopsis lyrata* (named as homolog-1 or HM2) (Accession no, CAC37558.1 and XP_020891373.1) with 98% identity.

### 2.2. Molecular dynamic simulations of the RsAFP2 protein, its two mutants and two homologous proteins

The RsAFP2 protein was downloaded from the PDB database (PDB ID, 2n2r) and two homologs; HM1 and HM2 were generated using Swiss model server with 2n2r as template (sequence identity: 98.04%) which was used to prepare the initial topology parameter files using *tleap* of AmberTools18. The system was solvated in TIP3P water which added 4587 water residues with the final box size of 62.921 × 55.225 × 54.498 Å^3^. Similarly, the two mutants, G9R and V39R were prepared using PyMol and then solvated in 4584 TIP3P water molecules with a system box size of 62.921 × 55.225 × 54.498 Å^3^ and 4635 water molecules with a system box size of 62.921 × 55.536 × 54.498 Å^3^ respectively. The two homologous proteins were solvated with 4662 water molecules in total box size of 63.118 × 55.225 × 54.671. All systems were made to undergo a six-step minimization and equilibration procedure to prepare for accelerated molecular dynamics simulations as described by [37] followed by short classical MD (cMD) production run to obtain parameters for aMD. Subsequently, for each case, two parallel 500 ns long accelerated MD simulations were run from different starting structures.

All aMD simulations were carried out at 300 K temperature, 2 fs time step, and energies and boost information were recorded at every 1000 steps. The electrostatic interactions were calculated using PME (particle mesh Ewald summation) [38] and long-range interactions were also calculated with cutoff of 10.0. The temperature scaling was carried out using Langevin thermostat without pressure scaling. SHAKE algorithm was applied on all bonds involving hydrogen. The GPU machines available through the NIIF clusters of Hungary were utilized using *pmemd*.*cuda* implementation of Amber14. aMD can be carried out using three criteria, i) the whole potential at once (iamd = 1) or ii) independently boosting the torsional terms of the potential (iamd = 2), and iii) to boost the whole potential with an extra boost to torsions (iamd = 3). The third criterion seemed to be an appropriate choice, as the dual boost option provides a better reweighting distribution. The extra parameters E_dihed_, α_dihed_, E_total_ and α_total_ were calculated as required in Eq (1):

$$\begin{array}{l} {\text{E}}_{\text{dihed}}={\text{V}}_{\text{avg}\_\text{dihed}}+{\text{a}}_{\text{1}}\times {\text{N}}_{\text{res}},\;\alpha_{\text{dihed}}={\text{a}}_{\text{2}}\times {\text{N}}_{\text{res}}/5;\\ {\text{E}}_{\text{total}}={\text{V}}_{\text{avg}\_\text{total}}+{\text{b}}_{\text{1}}\times {\text{N}}_{\text{atoms}},\;\alpha_{\text{total}}={\text{b}}_{\text{2}}\times {\text{N}}_{\text{atoms}} \end{array}$$ (1)

Where N_res_ is the number of peptide residues (21, with an addition of acetyl group at the N-terminal), N_atoms_ is the total number of atoms in the system. V_avg_dihed_ and V_avg_total_ are average dihedral and total potential energies obtained from the cMD run. The various parameters used for all aMD simulations have been summarized in Table 1 based on coefficient values of a1, a2 as 4 and b1, b2 as 0.16. The structures were visualized using UCSF Chimera 1.12 [39].

**Table 1:** Summary of various accelerated molecular dynamics parameters.

Two parallel simulations of a single protein	E_total_ (kcal·mol^−1^)	α_total_	E_dihed_ (kcal·mol^−1^)	α_dihed_	Average boost (kcal·mol^−1^)
RsAFP	-42773	2325	861	40.8	11.21
G9R	-42903	2325	853	40.8	11.10
V39R	-43395	2325	851	40.8	29.85
Homolog-1	-43474	2362	845	40.8	17.50
Homolog-2	-43486	2325	843	40.8	17.23
Fungal membrane simulations					
RsAFP	-40543	3734	NA	NA	21.13
G9R	-41321	3936	NA	NA	23.12
V39R	-41891	3992	NA	NA	24.46
Homolog-1	-41258	3927	NA	NA	22.62
Homolog-2	-41166	3928	NA	NA	23.65

### 2.3. Preparation of RsAFP2 protein and its two mutants embedded within a fungal membrane mimic bilayer

All three systems were prepared using the CHARMM-GUI server’s Membrane Builder utility (http://www.charmm-gui.org). The proteins were aligned along the Z-axis (perpendicular to the bilayer normal) in such a way that the largest area covered by the protein lies in center of the Z-axis. A model fungal membrane mimic consisting four different types of phospholipids: 1-palmitoyl-2-oleoyl-sn-glycero-3-phosphocholine (POPC), 1-palmitoyl-2-oleoyl-sn-glycero-3-phosphoethanolamine (POPE), 1-palmitoyl-2-oleoyl-sn-glycero-3-phosphoserine (POPS), and 1-palmitoyl-2-oleoyl-sn-glycero-3-phosphoinositol-3-phosphate (PIP1) was used as a fungal membrane model. PIP1, termed as POPI13 in CHARMM under the phosphoinositol class. The ratio used for POPC: POPE: POPS: POPI13 is 5:3:1:1 as taken from [40]. Based on the same study, 40% of fungal sterol component, Ergosterol was also added to all systems. The system size was set to be 55.166, 55.166 and 85 along the X, Y and Z directions respectively. The number of lipids packed on both leaflets is 60. Along with the water box, 22 K^+^ and 12 Cl^-^ ions were added to neutralize the system (0.15 KCl system default). The GUI results in Amber-friendly topology format files. The three systems were prepared for aMD simulations using the same six steps as mentioned above. After a short production run, the aMD parameters were calculated as mentioned in Table 1. All simulations were run for 200 ns with boost to potential energy term only under the same conditions as mentioned in the previous sections. The simulations were analyzed using Membrane Analysis Tool or MEMPLUGIN available through Visual Molecular Dynamics (VMD).

### 2.4 Molecular dynamic trajectory analysis

Secondary structure analysis was performed using STRIDE [41], and Visual Molecular Dynamics (VMD) [42]. The representative structures were subjected to RING 2.0 for residue network analysis of the wild-type and mutant proteins [43].

### 2.5. Computation of free energy change

The change in the free energy (ΔΔG) between wild-type and mutant proteins was computed using the FoldX [44]. The difference in free energy (ΔΔG) is given by:

ΔΔG=ΔGmut-ΔGwt (2)

## 3. Results and discussion

Plants are exposed to a wide range of pathogenic fungi, and to combat, plants have evolved defense mechanisms that include the use of low molecular weight proteins that possess antifungal activity. Among the various classes of antifungal proteins, defensin is a cysteine-rich protein found in plants, fungi, insects, and mammals [17, 45, 46]. Long MD simulations were performed to examine the changes in folding pattern, conformation, and flexibility of the antifungal peptide RsAFP2 after amino acid substitutions at two positions.

### 3.1. Multiple sequence alignment of RsAFP2

**Fig 1:**
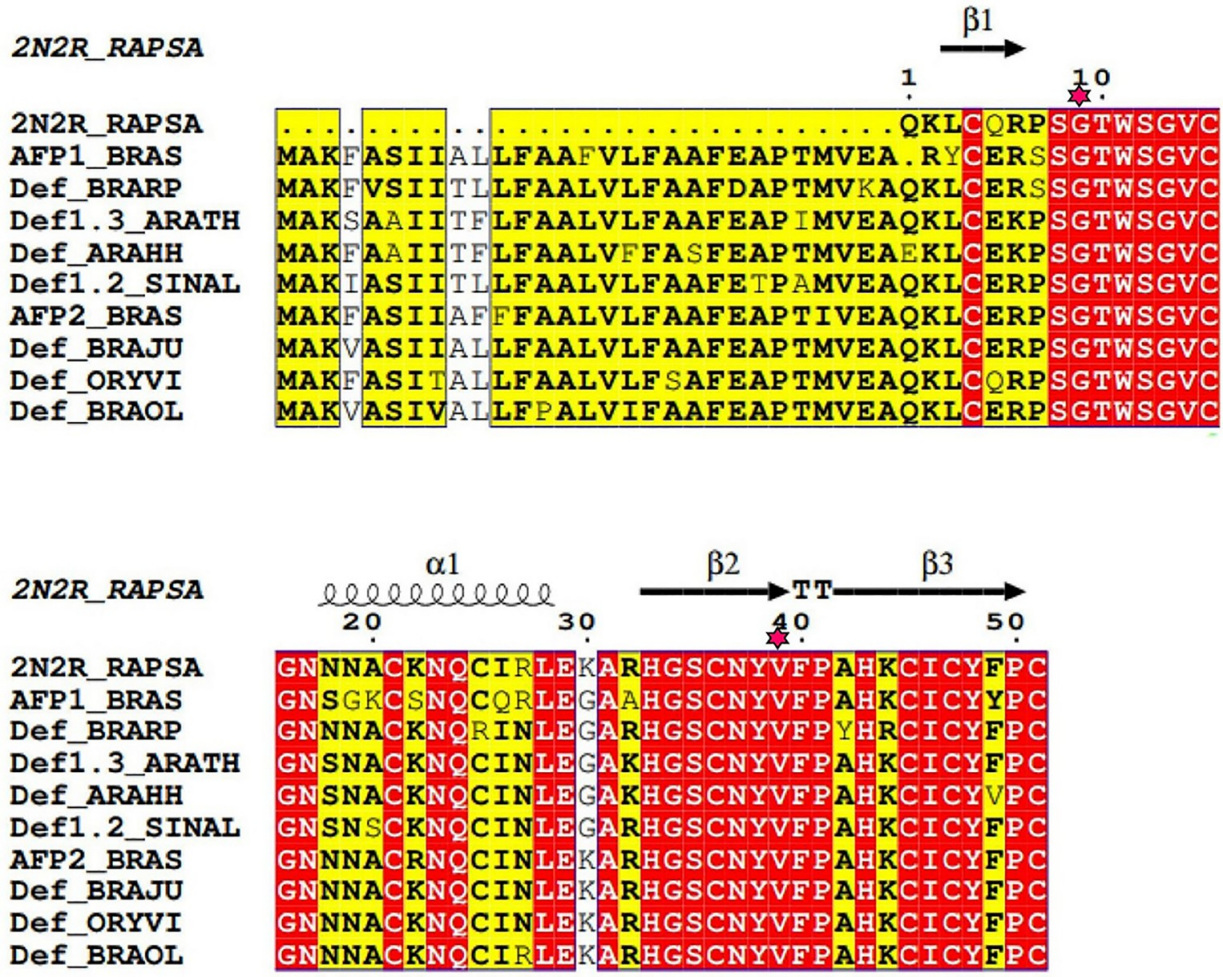
Multiple sequence alignment of homologous RsAFP2 proteins from different species. Red columns indicate conserved residues with in the alignment, and red stars indicate the 9th and 39th positions in the alignment. **Abbreviations** RAPSA: *Raphanus sativus*; ARATH: *Arabidopsis thaliana*; ARAHH: *Arabidopsis halleri* subsp. halleri; BRARP: *Brassica rapa* subsp. pekinensis; BRAS: *Bradyrhizobium* spp.; SINAL: *Sinapis alba*; BRAJU: *Brassica juncea*; ORYVI: *Orychophragmus violaceus*; BRAOL: *Brassica oleracea*.

Multiple sequence alignment of the RsAFP2 proteins and selected homologous defensin proteins from different plants species revealed a high level of conservation in the sequence and secondary structural of these proteins [47]. The residues at the 9^th^ and 39^th^ positions (numbering according to radish) were found to be strictly conserved across the alignment (Fig 1). The eight cysteine residues at positions Cys4, Cys15, Cys21, Cys36, Cys45, Cys47, and Cys51 were found to be highly conserved, with the exceptions of Cys25, in *Brassica rapa*. Cys25 was found to be substituted to arginine (Fig 1). These findings indicate that these residues are subjected to considerably strong selection pressure, as they play a critical role in the detection of various fungal species. The RsAFP2 peptide contains the residues Leu3-Arg6, His33-Val39, and Ala42-Pro50 within β strand conformations, Asn18-Leu28 within its α-helix, Ser8-Trp11 and Val39-Ala42 within its β-turns, His33-Val39 within its β hairpin, and residues 4–51, 15–36, 21–45, and 25–47 form disulfide bridges (Fig 1). These mutants were subjected to MD simulations to examine the conformational transitions that occurred from the beginning (t = 0 ns) to the end of the simulation run (t = 500 ns).

### 3.2 Accelerated molecular dynamics simulation analysis

**Fig 2:**
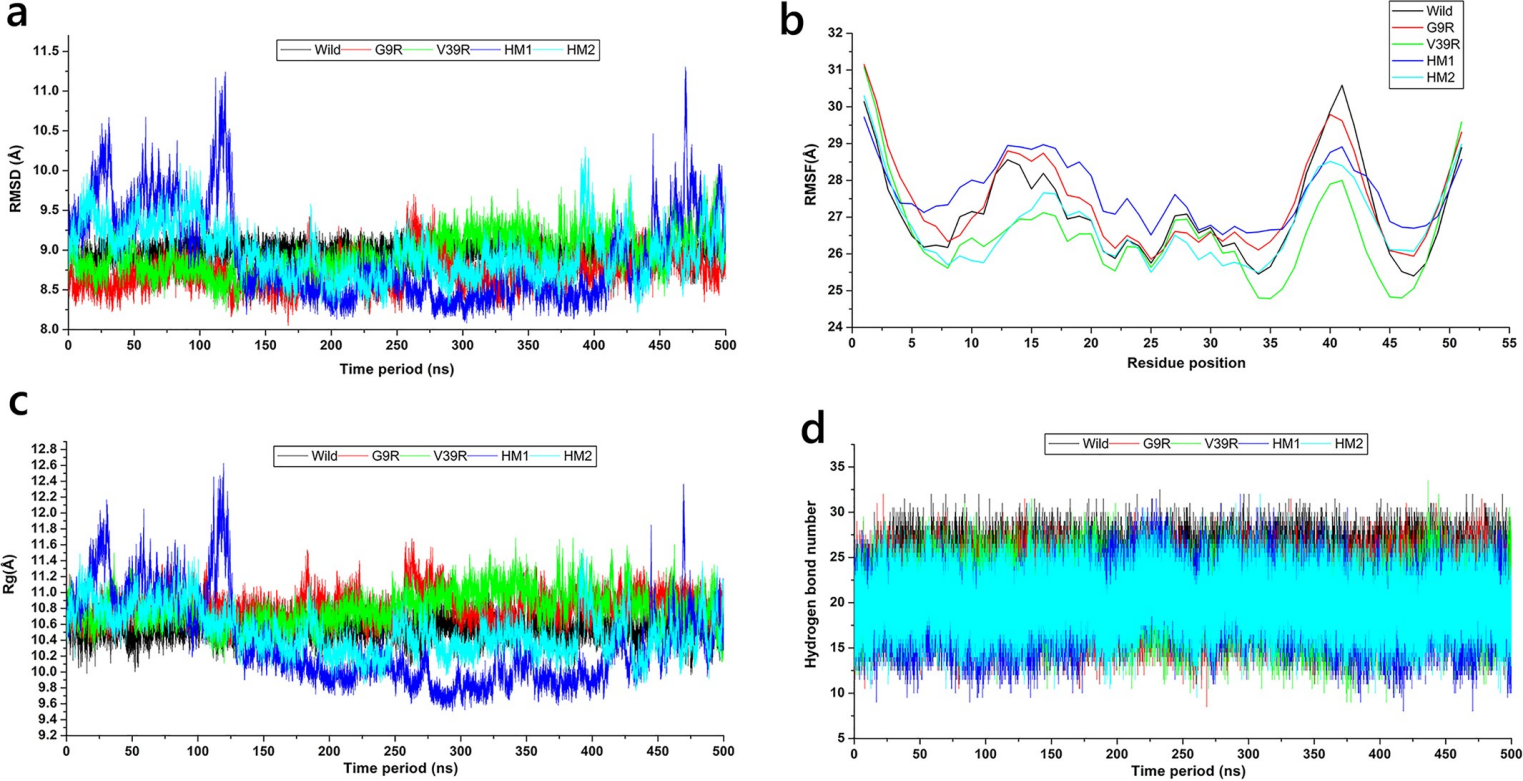
Molecular dynamics trajectory analysis. (a) Root mean square deviation (b) Root mean square fluctuation (c) Radius of gyration (d) Number of hydrogen bond for 500 ns MD simulations run.

Structural analysis was carried out from the average trajectory profile of two parallel MD runs. The stability of the RsAFP2 wild-type, mutant and homologs was determined using the root mean square deviation (RMSD) method from the two parallel MD simulations. The plot revealed that the convergence of the molecular dynamics RMSD trajectories for wild-type, mutants and homologs occurred at 450 ns, with an average RMSD of 9.0Å, 8.7Å, 8.9Å, 8.9Å, 9.0Å for wild-type, G9R, V39R, HM1 and HM2, respectively (Fig 2A). The substitution of hydrophobic, non-charged residues with hydrophilic, charged arginine residues confers greater stability in comparison to that of wild-type. The RMSD was analyzed every 100 ns from t = 100 ns to t = 500 ns during the MD simulations for the wild-type, mutants and homologs (Table 2).

**Table 2:** RMSD of RsAFP2 wild-type and mutant proteins at different time intervals.

System	RMSD (Å)				
	100 ns	200 ns	300 ns	400 ns	500 ns
Wild-type	9.1	8.9	9.0	9.1	9.2
G9R	8.6	8.5	8.6	8.8	9.0
V39R	8.8	8.7	9.1	9.4	9.2
HM1	9.2	8.4	8.3	8.6	9.5
HM2	9.4	8.5	8.7	9.3	9.2

Root mean square fluctuation (RMSF) of the backbone atom of the 51 amino acid residues examined in the defensing protein revealed that the residues at positions 35–40 exhibited the highest fluctuations in all the systems (Fig 2B). The average RMSF for the wild-type, G9R, V39R, HM1 and HM2 was 27.15Å, 27.44Å, 26.54Å, 27.65Å and 26.80Å respectively. The RMSF of residues Gly9 and Val39 in the wild-type was 27 Å and 29 Å, respectively, while those values for G9R and V39R were 26.5 Å and 27.4 Å, respectively. All the cysteines, however, were found to possess similar flexibility that of the wild-type (Table 3). The radius of gyration was computed for the wild-type and mutants to measure the differences in protein compactness during the simulation run. The average Rg scores for the wild-type, G9R, V39R, HM1 and HM2 were found to be 10.6Å, 10.8Å, 10.8Å, 10.4Å, 10.5Å, respectively (Fig 2C). Hydrogen bonding (H-bond) for the wild-type, G9R, V39R, HM1 and HM2 was shown in Fig 2D.

**Table 3:** RMSF of critical residues in wild-type and mutants at different time intervals.

Residues	RMSF (Å)				
	Wild-type	G9R	V39R	HM1	HM2
Gly9	27.0	26.5	26.2	27.8	25.9
Val39	29.0	29.2	27.4	28.2	28.4
Cys4	27.1	28.1	27.5	27.4	27.2
Cys15	27.8	28.5	26.9	28.8	27.2
Cys21	26.1	26.5	25.7	27.2	26.1
Cys25	25.8	25.9	25.6	26.5	25.5
Cys36	26.3	26.7	25.0	26.7	26.2
Cys45	26.0	26.1	24.8	26.9	26.1
Cys47	25.4	25.9	25.1	26.7	26.1
Cys51	28.9	29.3	29.6	28.6	29.0

Solvent Accessible Surface Area (SASA) was computed for the wild-type and the mutants, and this analysis revealed increase in SASA value from the wild-type (166.4 Å^2^) to G9R (171.50 Å^2^). Similarly, an increase in the SASA value in V39R (171.25 Å^2^) in comparison to that of the wild-type (168.25 Å^2^) can be clearly observed in the SASA plot (S1 Fig). The increase in the SASA value was due to the substitution of a hydrophobic residue with a hydrophilic residue (arginine). The representative structures were extracted from the stable time frame (t = 450 ns to t = 500 ns) and overlapped with the native structure with the RMSD of 0.94Å, 1.3Å, 1.1Å, 1.24Å for wild-type, G9R, V39R, HM1 and HM2 respectively.

### 3.3. Total energy analysis of the systems and free energy change (ΔΔG) analysis

The representative structures for the RsAFP2 wild-type and mutant proteins were analyzed to determine the Born self-energy, coulomb energy, electrostatic solvation energy, and total energy. The results of these analyses indicated that the stability of the mutants was greater than that of the wild-type (Table 4). The total energy computed from MD simulations for the G9R, V39R, HM1 and HM2 was determined to be -34258.75 kJ/mol, -34659.66 kJ/mol, -34696.00 kJ/mol and -34705.78 kJ/mol, higher than that of the wild-type (-34133.40 kJ/mol; S2 Fig). The impact of the mutations on the peptide conformation and stability was predicted by computing the free energy change between the RsAFP2 wild-type and the mutant proteins. The free energy changes (ΔΔG) of 0.24 kcal/mol and -0.12 kcal/mol were observed between wild-type and G9R and wild-type and V39R, respectively, indicated that the mutations exerted a neutralizing and slight stabilizing effect on peptide stability based on the FoldX criteria [48].

**Table 4:** Energy analysis for the RsAFP2 wild-type and mutants.

Mutants	Born self-energy (kJ/mol)	Coulomb energy (kJ/mol)	Electrostatic solvation energy (kJ/mol)	Total energy (kJ/mol)
Wild-type	-2226.60	-13171.41	-286.16	-13018.16
G9R	-2271.11	-13671.16	-459.588	-13691.27
V39R	-2304.53	-13789.08	-376.35	-13698.24
HM1	-2199.27	-13365.40	-247.72	-13144.12
HM2	-2255.56	-13242.23	-321.29	-13070.80

### 3.4 Secondary and three-dimensional structure analysis

Secondary structure element (SSE) analyses were used to predict changes in the RsAFP2 wild-type and mutant proteins that occurred between 0 ns and 500 ns at 100 ns intervals. At 0 ns, the amino acid residues at the 9^th^ and 39^th^ positions existed within the turn and β strand structure in the wild-type; however, the 9^th^ and 39^th^ residues in G9R and V39R were located within the turn (Table 5). Additionally, during the simulation run, the β-strand comprising the V39 in the wild-type was changed to turn (Table 5). Additionally, total percentage of the secondary structure element revealed high percentage of β-sheet in the V39R relative to wild-type, other systems, with almost similar percentage of α-helix (S3 Fig). Three-dimensional structure conformations were also computed from 0 ns to 500 ns at 100 ns intervals for the wild-type and the mutant proteins (S4–S8 Figs and S1 Table).

**Table 5:** Variation in the secondary structure elements of RsAFP2 at different time intervals.

Systems	Time interval											
	0 ns		100 ns		200 ns		300 ns		400 ns		500 ns	
Wild-type	Turn	Strand	Turn	Turn	Turn	Strand	Turn	Turn	Turn	Turn	Turn	Turn
	(9)	(39)	(9)	(39)	(9)	(39)	(9)	(39)	(9)	(39)	(9)	(39)
G9R	Turn (9)		Turn(9)		Strand(9)		Strand (9)		Coil(9)		Coil(9)	
V39R	Turn(39)		Turn(39)		Turn(39)		Strand (39)		Turn(39)		Turn(39)	
HM1	Turn	Strand	Coil	Strand	Turn	Turn	310Helix	Turn	Turn	Turn	Turn	Turn
	(9)	(39)	(9)	(39)	(9)	(39)	(9)	(39)	(9)	(39)	(9)	(39)
HM2	Turn	Strand	Strand	Coil	Turn	Turn	Coil	Turn	Helix	Turn	Turn	Turn
	(9)	(39)	(9)	(39)	(9)	(39)	(9)	(39)	(9)	(39)	(9)	(39)

(9) and (39) are position in the peptide.

### 3.5. Intra-atomic interactions

Intra-atomic interactions in the wild-type, G9R, and V39R RsAFP2 proteins were computed from their representative structures. Residues at the 9^th^ and 39^th^ positions in the wild-type protein were not involved in any interactions. Increases in the intra-atomic interactions in the V39R RsAFP2 proteins could be responsible for their increased stability. A complete list of interactions is provided in S2–S6 Tables.

### 3.6. Results from aMD simulation of RsAFP2, its two mutants and two homologs with fungal membrane mimics

The membrane properties are modulated by the presence of trans-membrane proteins. In this case, RsAFP2, its two mutants and two homologs were simulated within fungal membrane mimic bilayer patches. The change in membrane characteristics was studied based on area-per-lipid at the bilayer interface with water calculated for each lipid as a function of simulation time, lipid order parameters, and thickness dependent disorder of the system. The Deuterium Order Parameter or S_CD_, is generally derived from through NMR experiments and shows the orientation mobility of each C-H bond along lipid tails (acyl chains) as a measure of membrane fluidity.

It is defined as,

S=<(3cos2Q-1)/2>

Where Q is the (time dependent) angle between the C–D bond vector and a reference axis. The angular brackets denote a time and ensemble average [49].

**Fig 3:**
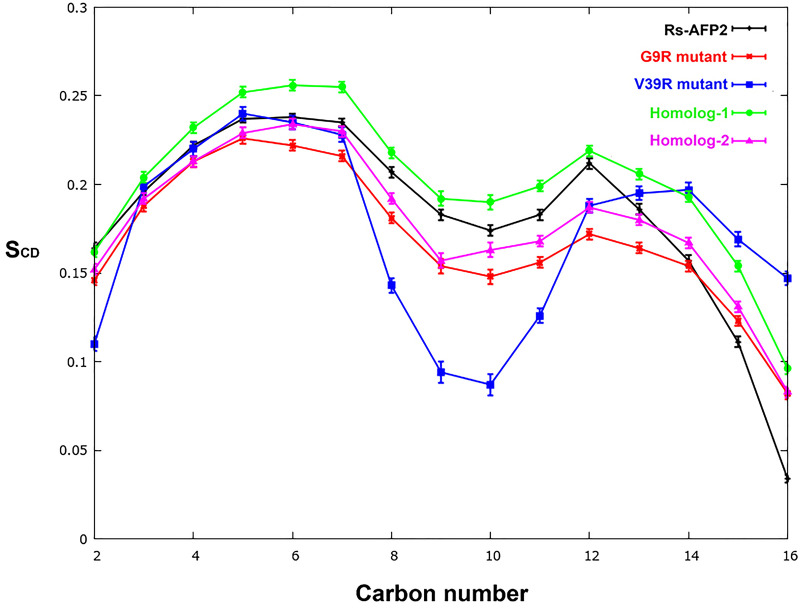
The deuterium order parameter, S_CD_, calculated for the sn-1 and sn-2 lipid acyl chains from all five systems comprising a wild-type RsAFP2, its two mutants and two homologs embedded in fungal mimicking bilayer membrane.

It is clear from the comparative values of order parameter that mutant V39R behaves very differently in introducing disorder to the membrane system (Fig 3). The averaged deuterium order parameter S_CD_, decreases abruptly for 9^th^ and 10^th^ carbons in the acyl chains of constituting lipids. The same effect is shown by G9R mutant but to a lesser extent while the native RsAFP2 and its two homologs do not bring such an extent of disorder to the membrane. The decrease in order parameter can be visualized as tilting of lipid acyl chains that leads to decrease in value of calculated membrane thickness. We also calculated the per-lipid average order parameters for in case of each protein variant and provided in S9 Fig. The S_CD_ parameters can be directly compared with those of provided in Ermakova et al. [40] for the M3 membrane system containing 40% ergosterol. The M3 membrane system shows a very high degree of order due to the presence of ergosterol especially in comparison to M1 and M2 membrane systems with no or 2% of ergosterol present, respectively. However, the addition of protein to these membranes increases disorder as can be seen that the S_CD_ parameter for most carbons of the sn1 and sn2 acyl chains lies above 0.3 while the membrane lipids in our systems lie under 0.25. The addition of the G9R mutant to this membrane brings further disorder in the POPC lipids. The V39R mutant, on the other hand, had the biggest effect on decreasing membrane order of the POPI lipids while its presence increases order of the POPS lipids. The biggest increase in order of POPS lipids could be observed in case of the homologs, 1 and 2 which is comparable to the M3 membrane system S_CD_ parameters.

Psd1, a pea defensin protein, was shown to have specificity towards membranes containing ergosterol and some glycosphingolipids [50]. Similarly, Psd2, was reported to show specificity towards *Fusarium solani* GlcCer and ergosterol along with POPC and some phosphatidylinositol species (for e.g. POPI) [51]. Upon comparing our results, we see that the G9R and V39R mutants bring significant difference in the order parameters of POPC and POPI lipids while POPS lipids are also affected by increase in order.

**Fig 4:**
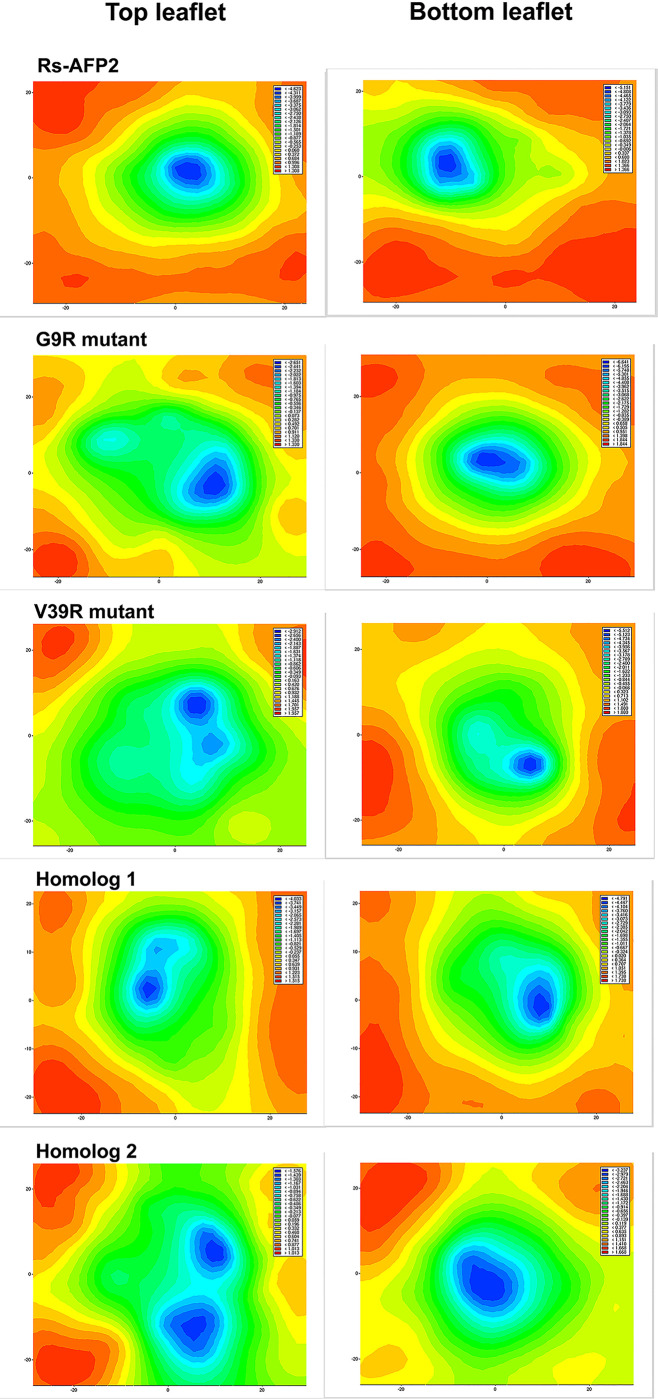
The 2D-deformation maps calculated from bilayer thickness for each protein-membrane system comprising a wild-type RsAFP2, its two mutants and two homologs. The darkest blue regions define the thinnest regions of the membrane owing to leakage of water molecules across the trans-membrane region. The red areas are indicating thickest membrane regions.

A 2D thickness/deformation map is generated for the top and bottom leaflets of the membrane that defines local thickness (Fig 4). *Deformation* is defined as the interpolated location of the head-groups after translating the chosen leaflet's center at Z = 0. Therefore, *deformation* takes on both negative and positive values. The positive values stand for membrane expansion while negative values otherwise. The red regions on the contour plot show maximum bilayer thickness. The deformation values for the native RsAFP2 lies between -4.6 to 1.3 for the top leaflet and -5.1 to 1.3 for bottom leaflet. For the G9R mutant these values are from -2.6 to 1.3 for top and -6.6 to 1.8 for bottom and for the V39R mutant these values are from -2.9 to 1.9 for top and -5.5 to 1.8 for bottom. Similarly, these values for homolog-1 are from -4.0 to 1.5 for top and from -4.7 to 1.7 for bottom leaflet while for homolog-2 these values are from -1.5 to 1.0 for top and from -3.2 to 1.6 for bottom.

**Fig 5:**
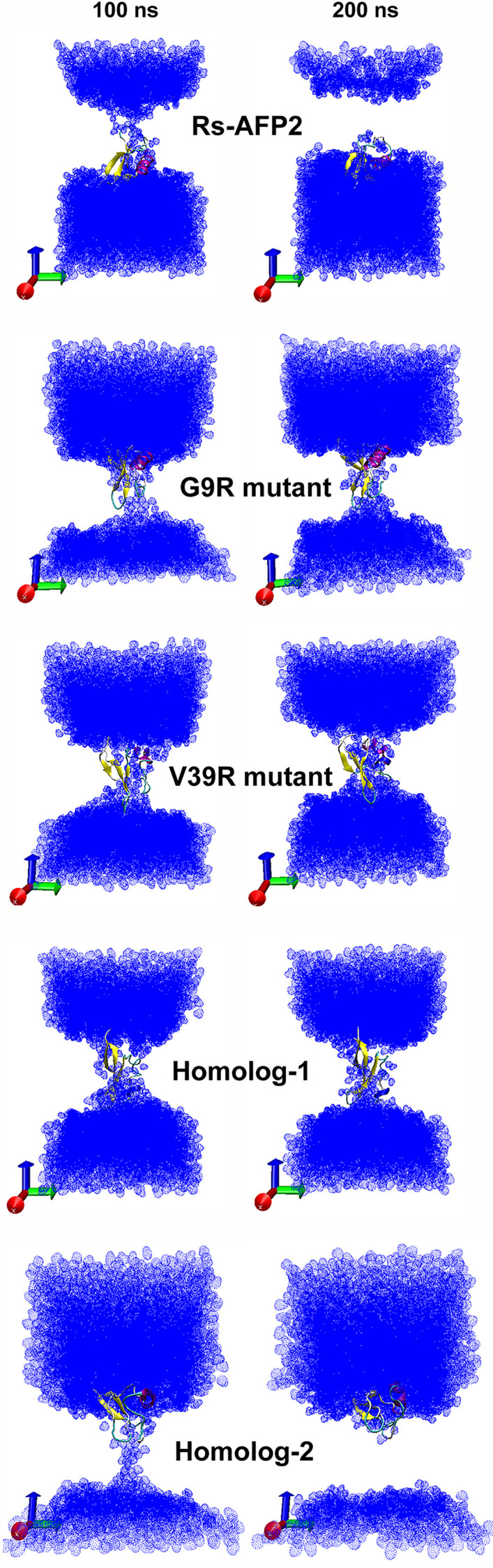
Snapshots at 100 and 200 ns of protein-membrane simulations for each system a wild-type RsAFP2, its two mutants and two homologs highlighting the extent of leakage of water molecules across the trans-membrane region. The two mutants cause higher influx of water molecules owing to the charged Arg residue replacement.

RsAFP2 contour plots show larger red regions while the two mutants show reduction in membrane thickness around protein placement. Upon comparison, the largest negative deformation values are observed for the bottom leaflet of the G9R mutant at -6.6 followed by the V39R mutant. At the top leaflet however, the native RsAFP2 has the highest negative value at -4.6 but the mutant variants show a larger area showing reduction in membrane thickness. This indicates that the mutations cause membrane thinning to a slightly larger extent in comparison to native RsAFP2. This can be explained by the increased permeability of water molecules through the bilayer. It could be clearly observed that the two mutants with the presence of an Arg side-chain could draw more water molecules across the bilayer, a phenomenon explained by the low energy cost of burying a charged amino acid like arginine [52]. The permeability of water across the trans-membrane region can be quantified using the average diffusion coefficient (DC cm^2^ s^-1^) calculated for water molecules displacing in the z-direction i.e. along the membrane normal. Displacement in the z-direction is indicative of membrane deformation caused by the protein. The DC value calculated for water in case of the native RsAFP2 protein in membrane is 0.0166 Χ 10^−5^ cm^2^ s^-1^ while for the G9R mutant is increases slightly to 0.0331 Χ 10^−5^ cm^2^ s^-1^ and for the V39R mutant it is the highest at 0.0354 Χ 10^−5^ cm^2^ s^-1^. This shows that the respective single mutations bring a slight increase in the diffusion of water across the bilayer and indicates higher deformation and activity of the anti-fungal protein variants. Similarly, the first and second homologous variants have DC values of 0.0220 Χ 10^−5^ cm^2^ s^-1^ and 0.0213 Χ 10^−5^ cm^2^ s^-1^ respectively, which is slightly higher than the native but less than the mutant proteins. The ability to accelerate water movement through the bilayer can be directly visualized as membrane deformation (refer Fig 5 with two screenshots for each system at 100 ns and at 200 ns). It is clear that the loop regions containing the mutated Arg residue in each case directly faces and interacts with water molecules. The same could be observed for the two homologs. RsAFP2 differs from its homologs at residue 5^th^ where glutamine residue is replaced by the negatively charged ion glutamic acid. This switch increases the membrane permeability value for homologs in comparison to RsAFP2.

**Fig 6:**
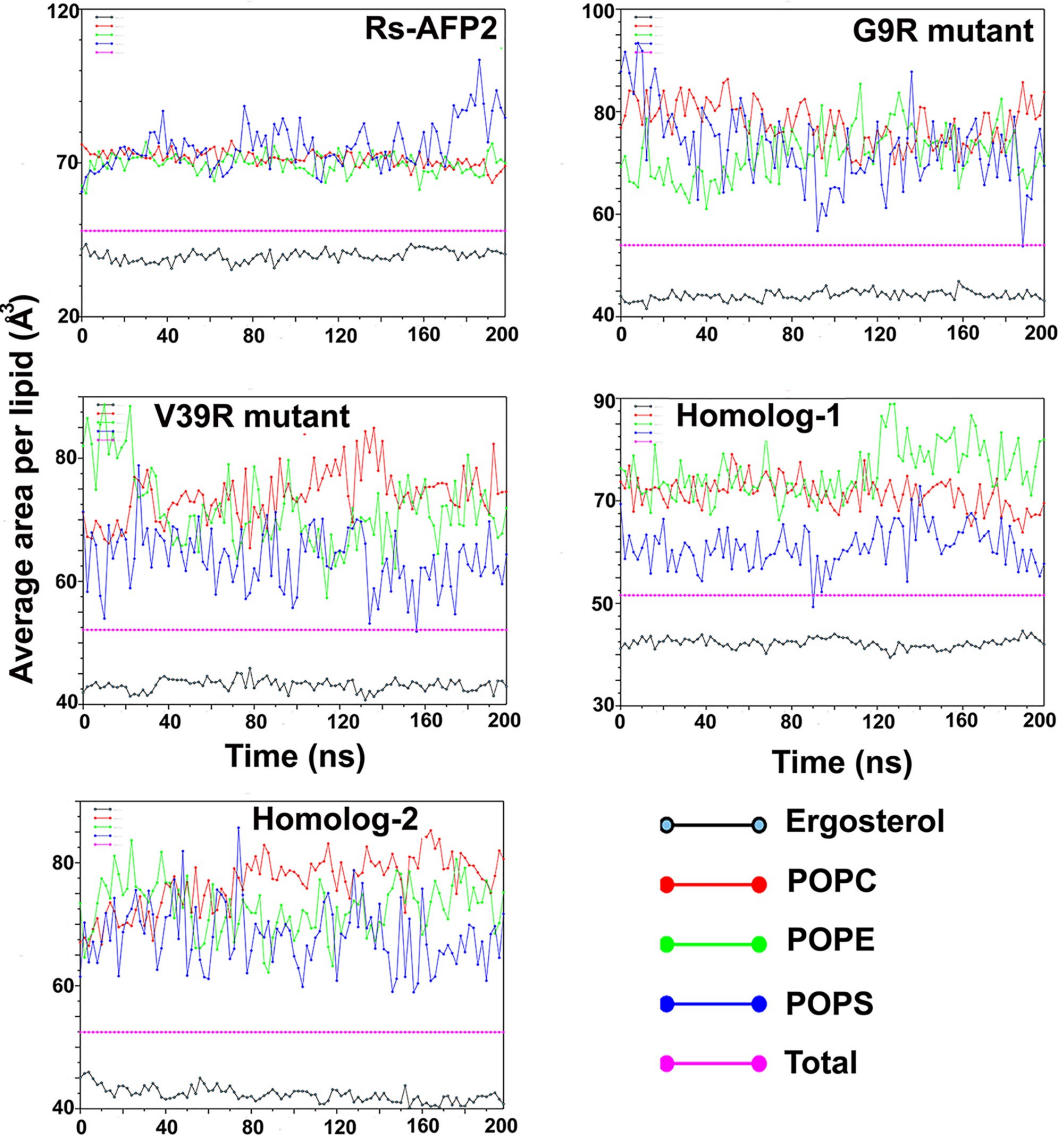
The area-per-lipid profiles of every constitutive lipid for each protein-membrane system comprising a wild-type RsAFP2, its two mutants and two homologs. Increasing area-per-lipid profiles increased membrane deformation. The profile remains very stable for RsAFP2 in comparison to its mutants suggesting that the mutation bring higher deformity in the fungal membranes.

Another popular method to estimate membrane disintegration is area-per-lipid calculated as total area and for each constitutive lipid species. A considerable increase in the area-per-lipid quantity indicates deformation in membranes. The area per lipid profiles for each system clearly shows highly fluctuating values for all lipids in case of the two mutants in comparison to RsAFP2 (Fig 6). The most abundant phospholipid, POPC clearly shows an increasing trend in case of the two mutants with values lying at or more than 80 Å^2^ while in case of native RsAFP2, these values remain low at around at 70 Å^2^. No significant change in the values for ergosterol could be observed to compare the native RsAFP2 and its two mutants. Relating this with the results obtained from S_CD_ order parameter analysis reported in S9 Fig, we can conclude that the presence of RsAFP2 brings about membrane deformation and leakage and is further enhanced by the G9R and V39R mutants. The POPC lipid species seem to be the most affected by protein insertion and shows highest disorder. The V39R mutant seems to be more potent than the G9R variant proven by the higher value of DC of water and also the overall S_CD_ parameters calculated in Fig 3. It is expected as many prior studies show that the 38 and 39 positions in RsAFPs are crucial for membrane binding and antifungal activity.

## 4. Conclusions

Accelerated molecular dynamics (aMD) simulations captured the conformational changes for RsAFP2 wild type, G9R and V39 mutants. The total energy of the mutants was found to be lower than then the wild-type, suggesting that substitution of a non-polar side chain with an ionizable side chain of arginine increases conformational stability. Furthermore, increases in the intra-atomic interactions and negative free energy change in the V39R RsAFP2 proteins suggested its increased stability. Therefore, substitutions of the key residues are likely to be highly useful for fungal defense strategies and the results of our study have highlighted the possible impact of these mutations on peptide conformation and stability that can alter the antifungal potency of these proteins. Therefore, the study suggested that aMD simulations are powerful tool to study the time dependent dynamics of the proteins.

## Supporting information

S1 Fig Surface accessible solvent area for 500 ns molecular dynamics trajectory analysis for RsAFP2 and its variants. (DOCX)

S2 Fig Total energy analysis for RsAFP2 wild-type, G9R and V39R mutants and homologs during the MD simulations. (DOCX)

S3 Fig Percentage of secondary structure element for RsAFP2, mutants and homologs. (DOCX)

S4 Fig Snapshot of the three-dimensional structure for RsAFP2 every 100 ns from t = 0 ns to t = 500 ns during the MD simulations. (DOCX)

S5 Fig Snapshot of the three-dimensional structure for G9R mutant every 100 ns from t = 0 ns to t = 500 ns during the MD simulations. (DOCX)

S6 Fig Snapshot of the three-dimensional structure for V39R mutant every 100 ns from t = 0 ns to t = 500 ns during the MD simulations. (DOCX)

S7 Fig Snapshot of three-dimensional structure for HM1 every 100 ns from t = 100 ns to t = 500 ns during MD simulations. (DOCX)

S8 Fig Snapshot of three-dimensional structure for HM2 every 100 ns from t = 100 ns to t = 500 ns during MD simulations. (DOCX)

S9 Fig The per-lipid average order parameters, S_CD_, for each protein variant embedded in the fungal membrane mimic. (DOCX)

S1 Table Secondary structure elements of RsAFP2 at different time intervals. (DOCX)

S2 Table List of interatomic interactions in the RsAFP2 wild-type protein. (DOCX)

S3 Table List of interatomic interactions in the RsAFP2 G9R mutant protein. (DOCX)

S4 Table List of interatomic interactions in the RsAFP2 V39R mutant protein. (DOCX)

S5 Table List of interatomic interactions in the RsAFP2 HM1 homolog protein. (DOCX)

S6 Table List of interatomic interactions in the RsAFP2 HM2 homolog protein. (DOCX)
