# Supplementary material for: Analysis of mutations of defensin protein using accelerated molecular dynamics simulations
Source: PLoS One. 2020 Nov 30;15(11):e0241679. doi: 10.1371/journal.pone.0241679 (PMC7703945; doi:10.1371/journal.pone.0241679)
Supplement: S2 Fig — (DOCX) [file pone.0241679.s002.docx]

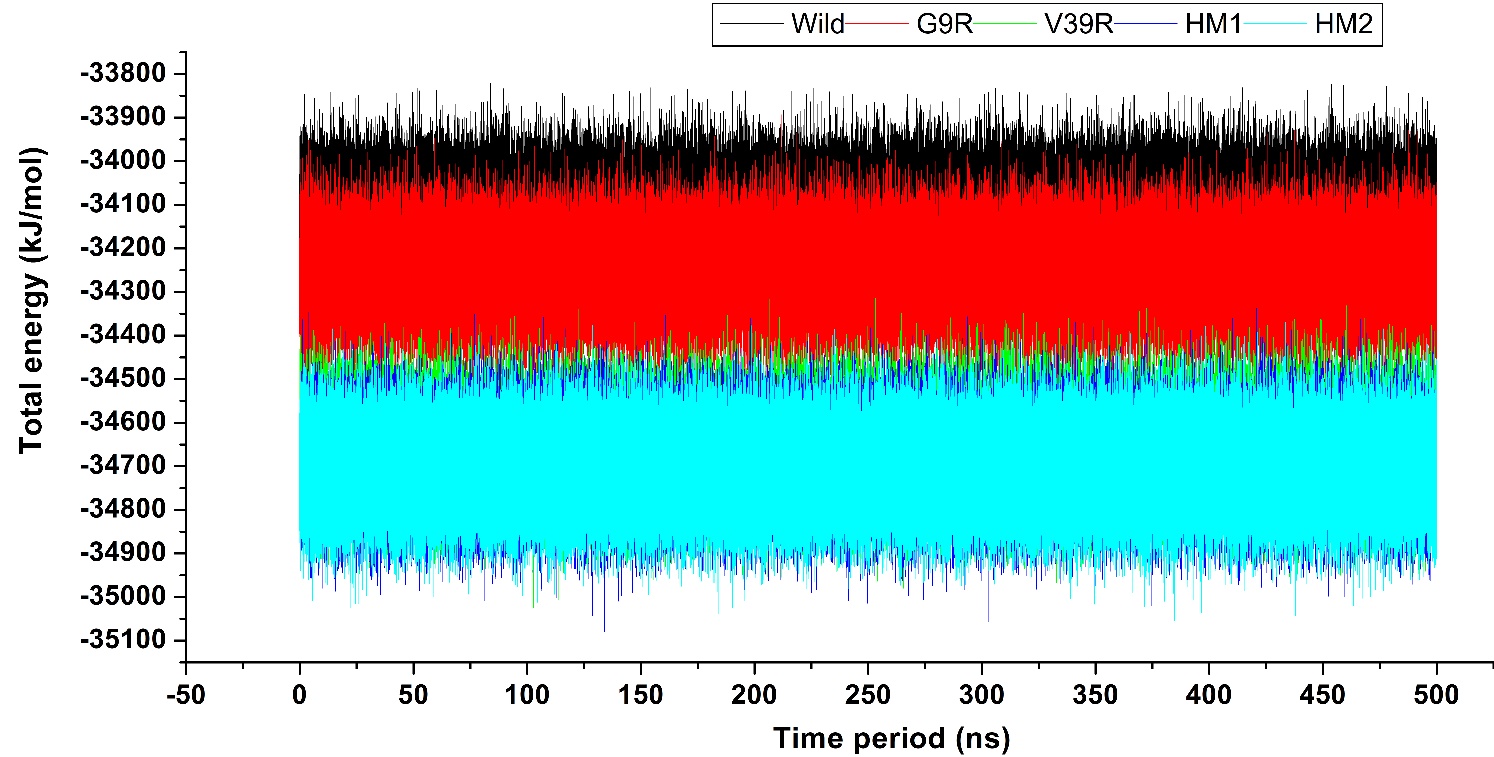


S2 Fig: Total energy analysis for RsAFP2 wild-type, G9R and V39R mutants and homologs during the MD simulations.
