# Supplementary figures and images for: Analysis of mutations of defensin protein using accelerated molecular dynamics simulations
Source: PLoS One. 2020 Nov 30;15(11):e0241679. doi: 10.1371/journal.pone.0241679 (PMC7703945; doi:10.1371/journal.pone.0241679)

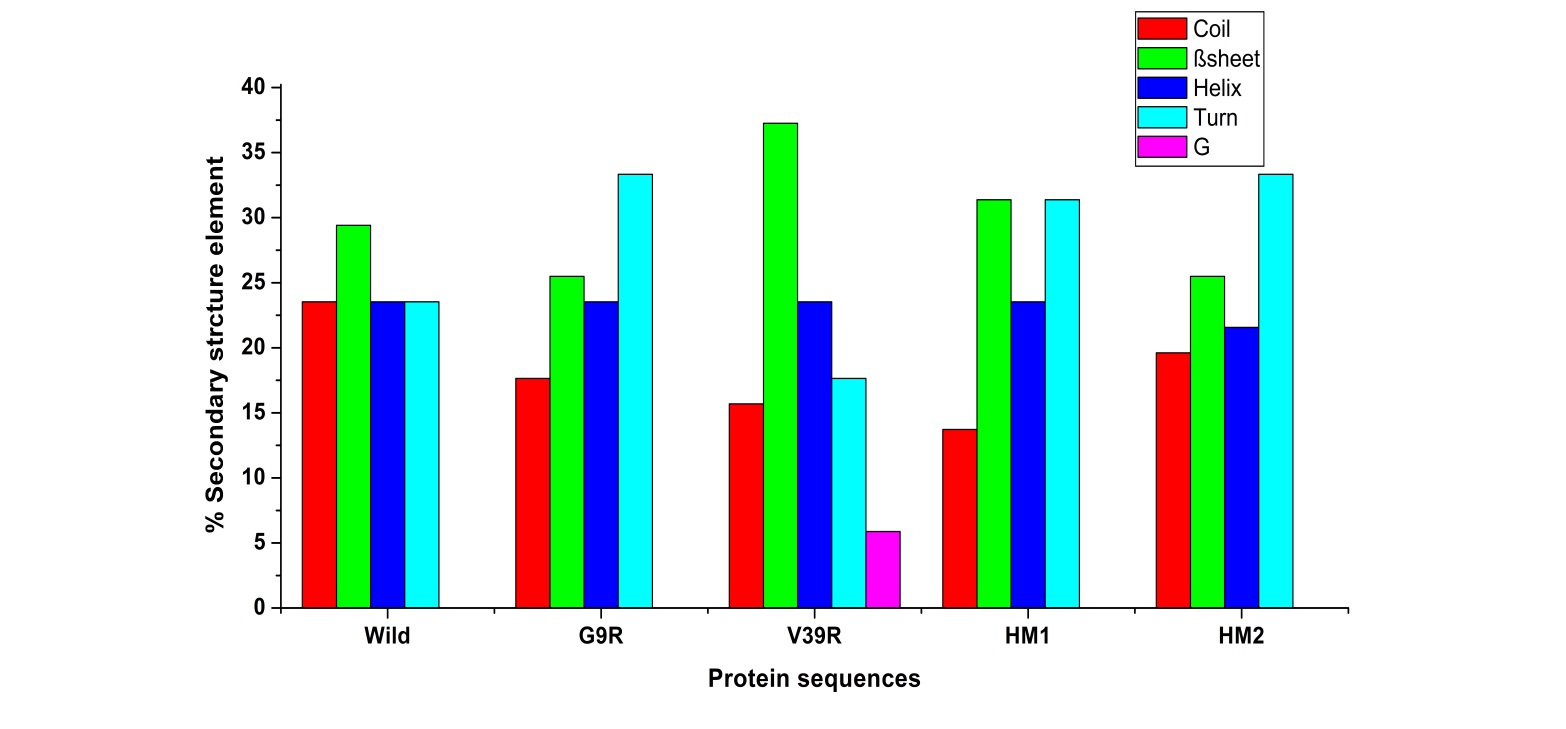


**S3 Fig:** Percentage of secondary structure element for RsAFP2, mutants and homologs.

Supplement: S3 Fig — (DOCX) [file pone.0241679.s003.docx]
