# Supplementary material for: Analysis of mutations of defensin protein using accelerated molecular dynamics simulations
Source: PLoS One. 2020 Nov 30;15(11):e0241679. doi: 10.1371/journal.pone.0241679 (PMC7703945; doi:10.1371/journal.pone.0241679)
Supplement: S6 Fig — (DOCX) [file pone.0241679.s006.docx]

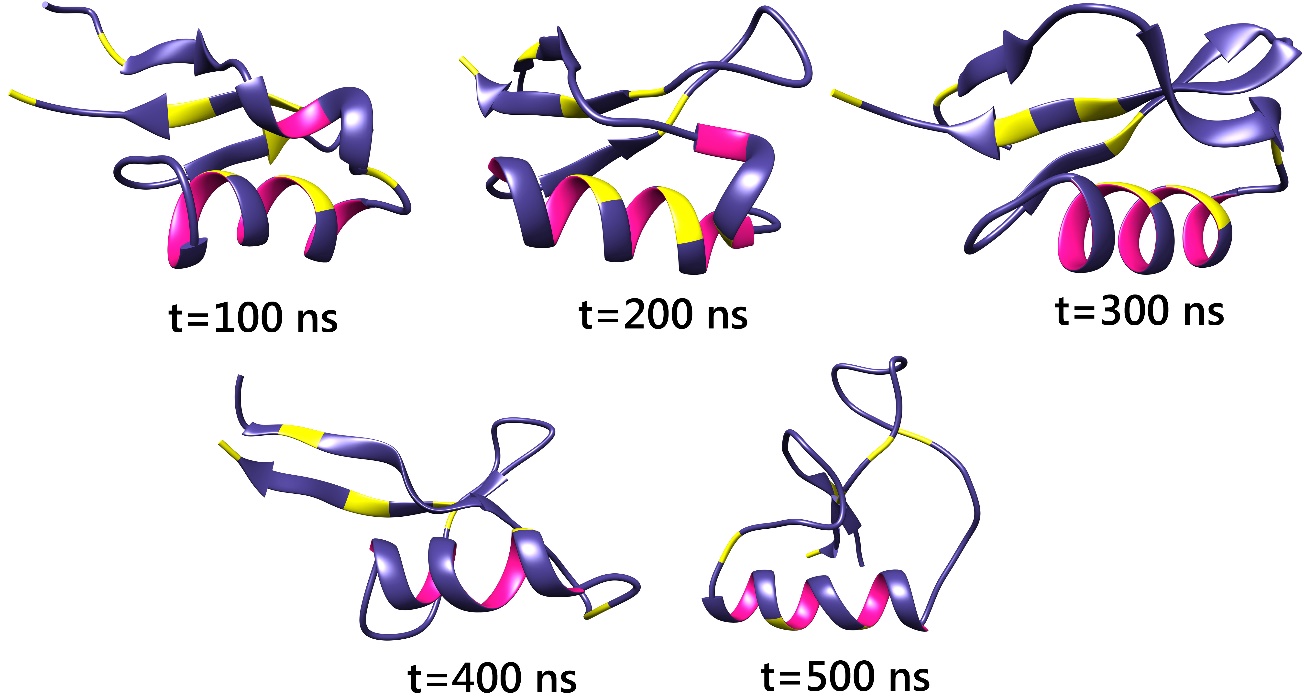


S6 Fig: Snapshot of the three-dimensional structure for V39R mutant every 100 ns from t = 0 ns to t = 500 ns during the MD simulations.
