# Supplementary material for: Analysis of mutations of defensin protein using accelerated molecular dynamics simulations
Source: PLoS One. 2020 Nov 30;15(11):e0241679. doi: 10.1371/journal.pone.0241679 (PMC7703945; doi:10.1371/journal.pone.0241679)
Supplement: S8 Fig — (DOCX) [file pone.0241679.s008.docx]

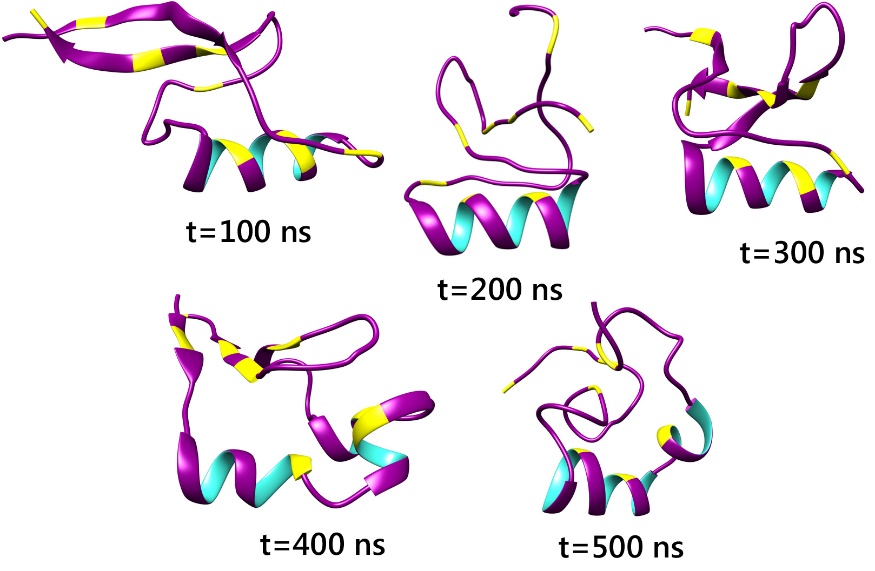


**S8 Fig**: Snapshot of three-dimensional structure for HM2 every 100 ns from t = 100 ns to t = 500 ns during MD simulations.
