# Supplementary material for: Analysis of mutations of defensin protein using accelerated molecular dynamics simulations
Source: PLoS One. 2020 Nov 30;15(11):e0241679. doi: 10.1371/journal.pone.0241679 (PMC7703945; doi:10.1371/journal.pone.0241679)
Supplement: S9 Fig — (DOCX) [file pone.0241679.s009.docx]

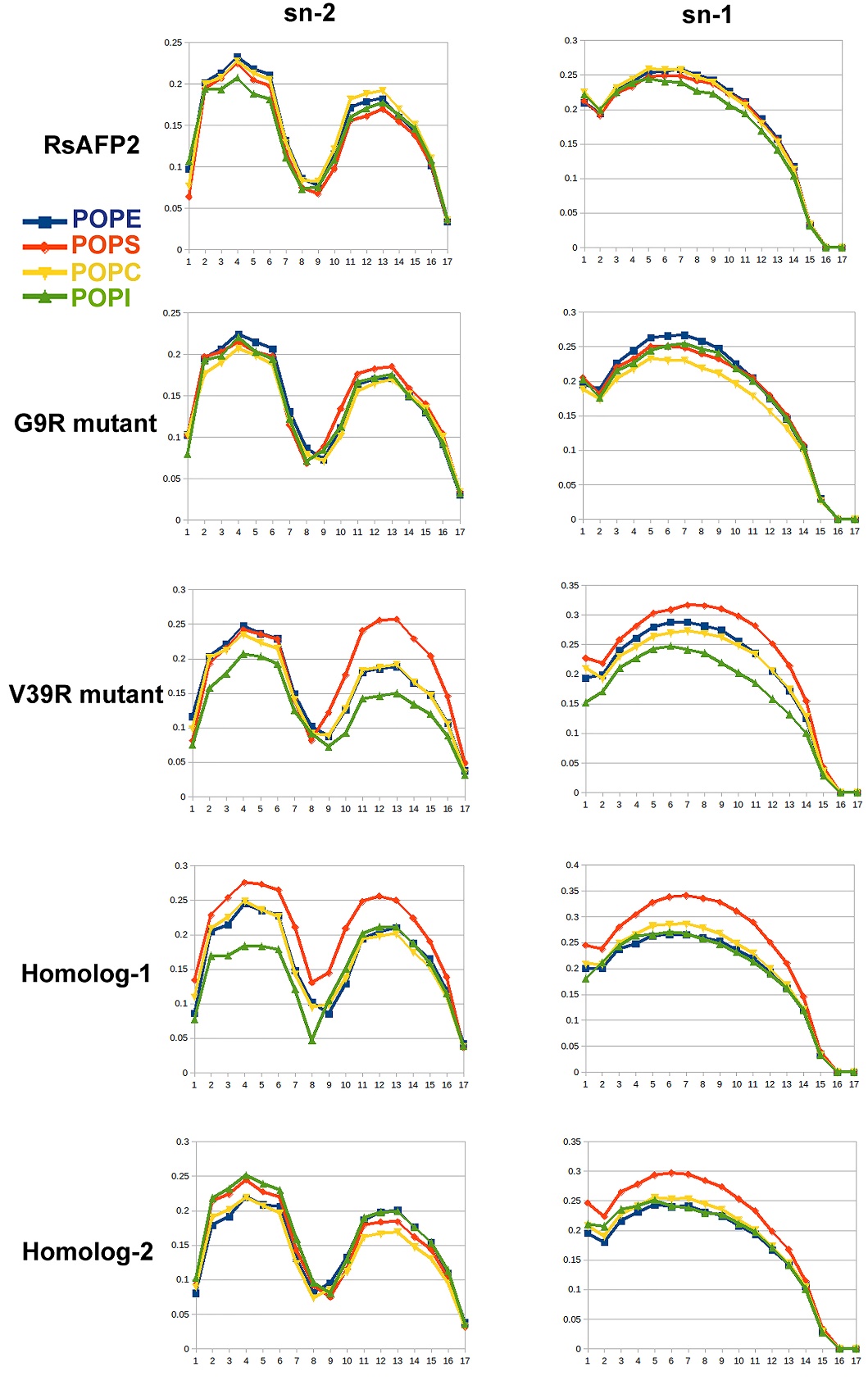


**S9 Fig.** The per-lipid average order parameters, S_CD_, for each protein variant embedded in the fungal membrane mimic
