# Supplementary material for: Analysis of mutations of defensin protein using accelerated molecular dynamics simulations
Source: PLoS One. 2020 Nov 30;15(11):e0241679. doi: 10.1371/journal.pone.0241679 (PMC7703945; doi:10.1371/journal.pone.0241679)
Supplement: S1 Table — (DOCX) [file pone.0241679.s010.docx]

**S1 Table:** Secondary structure elements of RsAFP2 at different time intervals

| **Systems** | **Time interval** | | | | | |
| --- | --- | --- | --- | --- | --- | --- |
|  | **0 ns** | **100 ns** | **200 ns** | **300 ns** | **400 ns** | **500 ns** |
| Wild-type | 3-6(E)  17-29(H)  33-39(E)  42-50(E) | 3-7 (E)  8-12(T)  13-17(C)  18-28(H)  33-37(E)  44-50(E) | 3-7(E)  8-12(T)  13-17(C)  18-28(H)  33-39(E)  42-50(E) | 3-7(E)  8-11 (T)  18-28(H)  33-37(E)  44-50(E) | 3-7(E)  8-11 (T)  18-28(H)  23-30(H)  33-37(E)  44-50(E) | 3-7(E)  8-11 (T)  18-28(H)  33-37(E)  44-50(E) |
| G9R | 2-6(E)  17-29(H)  33-39(E)  42-50(E) | 3-7(E)  18-29(H)  33-35(E)  37-43(T)  46-50(E) | 5-9(E)  10-19(T)  20-29(H)  33-37(E)  44-48(E) | 3-10 (E)  11-17(T)  18-29(H)  33-35(E)  44-50(E) | 3-10 (E)  11-17(T)  18-29(H)  33-35(T)  37-44(T)  46-50(E) | 3-7 (E)  11-17(T)  18-29(H)  33-34(E)  37-44(T)  46-50(E) |
| V39R | 2-6(E)  17-29(H)  33-39(E)  42-50(E) | 5-7(E)  8-17(T)  18-28(H)  33-36(E)  37-41(T)  45-48(E) | 3-11(E)  18-29(H)  33-36(E)  37-43(T)  44-50(E) | 5-14(E)  18-29(H)  34-39(E)  42-48(E) | 3-10(E)  19-28(H)  38-43(T)  44-50(E) | 3-5(E)  6-9(C)  17-29(H)  37-43(T)  48-50(E) |
| HM1 | 8-11(T)  18-29(H)  33-39(E)  42-50(E) | 3-8(E)  9-13(C)  18-28(H)  34-39(E)  42-50(E ) | 2-7(E)  9-13(T)  18-28(H)  34-36(E)  37-43(T)  44-49(E) | 7-9(310 Helix)  12-16(H)  18-28(H)  34-39(T)  41-44(T) | 8-16(T)  18-29(H)  31-34(T)  39-42(T) | 1-4(T)  7-9(T)  10-14(H)  18-28(H)  39-41(T) |
| HM2 | 8-11(T)  18-29(H)  33-39(E)  42-50(E) | 3-10(E)  18-29(H)  39-43(T)  44-50(E) | 8-12(T)  18-29(H)  32-36€  37-44(T) | 8-14(C)  18-29(H)  38-43(T)  44-49(E) | 8-16(H)  18-23(H)  25-28(H)  39-42(T)  45-49(E) | 2-11(T)  12-16(H)  19-25(H)  39-44(T) |
