# Supplementary material for: Analysis of mutations of defensin protein using accelerated molecular dynamics simulations
Source: PLoS One. 2020 Nov 30;15(11):e0241679. doi: 10.1371/journal.pone.0241679 (PMC7703945; doi:10.1371/journal.pone.0241679)
Supplement: S4 Table — (DOCX) [file pone.0241679.s013.docx]

S4 Table: List of interatomic interactions in the RsAFP2 V39R mutant protein.

| **V39R** | **Node1** | **Node 2** | **Distance(Å)** |
| --- | --- | --- | --- |
| HBOND:MC_MC | 14:_:VAL | 11:_:TRP | 3.1 |
|  | 43:_:HIS | 12:_:SER | 2.8 |
|  | 20:_:ALA | 17:_:ASN | 3.4 |
|  | 23:_:ASN | 19:_:ASN | 3.3 |
|  | 24:_:GLN | 20:_:ALA | 2.8 |
|  | 27:_:ARG | 23:_:ASN | 2.8 |
|  | 28:_:LEU | 24:_:GL | 3.1 |
|  | 29:_:GLU | 26:_:ILE | 3.3 |
|  | 30:_:LYS | 26:_:ILE | 2.9 |
|  | 31:_:ALA | 26:_:ILE | 2.8 |
|  | 48:_:TYR | 32:_:ARG | 3.2 |
|  | 48:_:TYR | 33:_:HIS | 3.1 |
|  | 46:_:ILE | 35:_:SER | 2.7 |
|  | 40:_:PHE | 37:_:ASN | 3.1 |

MC: Main chain, SC: Side chain, VDW: Van der Waals force, HBOND: Hydrogen bond
